# Detection and classification of unilateral cleft alveolus with and without cleft palate on panoramic radiographs using a deep learning system

**DOI:** 10.1038/s41598-021-95653-9

**Published:** 2021-08-06

**Authors:** Chiaki Kuwada, Yoshiko Ariji, Yoshitaka Kise, Takuma Funakoshi, Motoki Fukuda, Tsutomu Kuwada, Kenichi Gotoh, Eiichiro Ariji

**Affiliations:** 1 Department of Oral and Maxillofacial Radiology, Aichi Gakuin University School of Dentistry, Nagoya, Japan; 2 Division of Radiological Technology, Dental Hospital, Aichi-Gakuin University, Nagoya, Japan

**Keywords:** Medical research, Dental diseases, Oral anatomy

## Abstract

Although panoramic radiography has a role in the examination of patients with cleft alveolus (CA), its appearances is sometimes difficult to interpret. The aims of this study were to develop a computer-aided diagnosis system for diagnosing the CA status on panoramic radiographs using a deep learning object detection technique with and without normal data in the learning process, to verify its performance in comparison to human observers, and to clarify some characteristic appearances probably related to the performance. The panoramic radiographs of 383 CA patients with cleft palate (CA with CP) or without cleft palate (CA only) and 210 patients without CA (normal) were used to create two models on the DetectNet. The models 1 and 2 were developed based on the data without and with normal subjects, respectively, to detect the CAs and classify them into with or without CP. The model 2 reduced the false positive rate (1/30) compared to the model 1 (12/30). The overall accuracy of Model 2 was higher than Model 1 and human observers. The model created in this study appeared to have the potential to detect and classify CAs on panoramic radiographs, and might be useful to assist the human observers.

## Introduction

Cleft lip and palate (CLP) is one of the most common types of congenital maxillofacial lesions^1,2^. The frequency of babies born with CLP is approximately 1 in 500 in Japan^3^. Although various treatment protocols have been applied, soft tissue parts such as the lip and mucous membrane covering the hard plate are surgically treated within a relatively short period from birth^4^. Hard palatoplasty with bony transplant is frequently applied in patients with cleft alveolus (CA) to stabilize the maxillary segments, provide bony support for the teeth adjacent to the cleft, provide additional support to the lip and nose, and induce the canine eruption^5,6^. This surgery is usually performed at age 8–10 years, when the maxilla has grown sufficiently for the surgery. Therefore, patients need regular follow-up consisting of physical and imaging examinations for a long period. In this regard, panoramic radiography plays an important role in the repeated evaluation of cleft status because of its lower cost and radiation exposure levels than other modalities, such as computed tomography (CT) and cone-beam CT for dental use^7,8^. Focusing on the bony structures, patients with CA are divided into two types: CA with and without cleft palate (CP)^9^.

Cleft status including the classification can be easily identified by physical examination. However, for radiologists who interpret many radiographs and make many reports of their findings as routine work in a hospital radiology department, it is difficult to physically examine the patients. Therefore, radiologists have to estimate the patients’ status only based on radiographs. In this regard, panoramic radiography plays an essential role. However, it is sometimes difficult to interpret the images, especially for inexperienced radiologists, because of the overlap of the cervical spine and the narrow panoramic image layer, resulting in misdiagnoses as other radiolucent diseases and conditions, such as cysts, tumors, and fossa between the alveolar yokes of the central and lateral incisors. Moreover, even for experienced radiologists, it may be difficult to distinguish patients with CA with CP from those without CP. Therefore, if a computer-aided detection/diagnosis (CAD) system for the diagnosis of patients with CLP on panoramic radiographs can be established, it could greatly support such diagnosis, especially for inexperienced radiologists.

Deep learning (DL) algorithms using convolutional neural networks (CNNs) can detect and classify the features of certain objects, and they have been applied to CAD systems for diagnosis based on panoramic radiographs^10,11^. However, there have been no previous reports on such systems for patients with CLP. Some characteristic appearance features other than radiolucent areas directly indicating the CAs, on panoramic radiographs, such as the difference in the inferior line level of the piriform aperture and some findings of the maxillary lateral incisors of the affected side have been reported for patients with CA^12–16^, and they may be related to DL performance. Although a new technique to clarify the areas on which DL models focus has been introduced^17,18^, the findings contributing to DL performance are generally as unclear as a ‘black box’. Therefore, the above appearance features were analyzed in relation to the three groups (CA only, CA with CP and normal groups) in the present study.

The aims of the present study were to develop a CAD system for diagnosis of CLP on panoramic radiographs using a deep learning object detection technique with and without normal data in the learning process, to verify the DL model’s performance, and to clarify some characteristic appearance features probably related to the performance. Moreover, the usefulness of DL models was verified by comparing their performances with those by human observers.

## Material and method

Informed consent was obtained from all patients for being included in the study. This study was approved by ethics committee of Aichi Gakuin University (No. 496) and was performed in accordance with the tenets of the Declaration of Helsinki.

### Patients

The panoramic radiographs of 383 patients (169 female and 214 male) with unilateral CA that were acquired between August 2004 and July 2020 were selected from our hospital image database retrospectively. All patients were verified as having unilateral CA by medical records and CT or cone-beam CT examinations. The mean age of both male and female patients was 9.3 years. Of the 383 patients, 174 had solely CA and were assigned as the CA only group, whereas 209 had CA with CP and were designated as the CA with CP group. The CA only group was differentiated from the CA with CP group by referring to the patients’ medical records and CT images. Cases in which the cleft was limited to the anterior area of the incisive foramen on the most inferior axial CT slice, where the foramen was visible, were assigned to the CA only group. Cases in which the cleft extended posteriorly beyond the incisive foramen were assigned to the CA with CP group. Patients who had received surgical interventions for bony structures around the cleft before the first panoramic examination were excluded. In most patients, panoramic examinations were performed several times before bony transplant surgery. The panoramic images taken just before the transplant were selected for the present study. As controls, 210 panoramic radiographs matching the mean age and sex distributions of patients were selected from the same database during the same period. These patients, who were assigned as the normal group in the present study, were examined for other purposes, such as the evaluation of unerupted permanent teeth and pre-examination for orthodontic treatment.

The panoramic radiographs were exposed using an AUTO III NTR unit (Asahi Roentgen Industry, Kyoto, Japan), with a tube voltage of 75 kV, tube current of 12 mA and exposure time of 12 s or a Veraview Epocs unit (J. Morita Mfg. Corp., Kyoto, Japan), with the tube voltage of 75 kV, tube current of 8 mA and exposure time of 16.2 s.

### DL architecture

The DL system was created on Ubuntu Linux operating system version 16.04.2. The workstation had a GeForce 1080Ti GPU with 11GB of memory (NVIDIA, Santa Clara, CA). The deep learning process was performed using a customized DetectNet built in the Digits version 5.0 (NVIDIA, Santa Clara, CA; https://developer.ndivia.com/digits) training system. The Adam (adaptive moment estimation) solver was used for the training process with 0.0001 as the base learning rate. DetectNet has five main parts: data ingestion and augmentation, a fully convolutional network, loss function measurement, bounding box clustering, and mean average precision calculation^19^.

### Development of learning models

Two models (models 1 and 2) were created. The panoramic radiographs were downloaded from the database in JPEG format, and all images were cropped to 900×900 pixels. In each group (including the CA only, CA with CP, and normal groups), 30 images were randomly assigned to test dataset, and the remaining images, which included the training (approximately 80% of the remaining data) and validation datasets, were used to create the learning models (Table 1).

**Table 1:** Summary of datasets (number of panormaic images).

Group	Dataset			
	Training	Validating	Testing	Total
CA only	115	29	30	174
CA with CP	143	36	30	209
Normal	144	36	30	210
Total	402	101	90	593

*CA* cleft alveolus, *CP* cleft palate.

**Figure 1:**
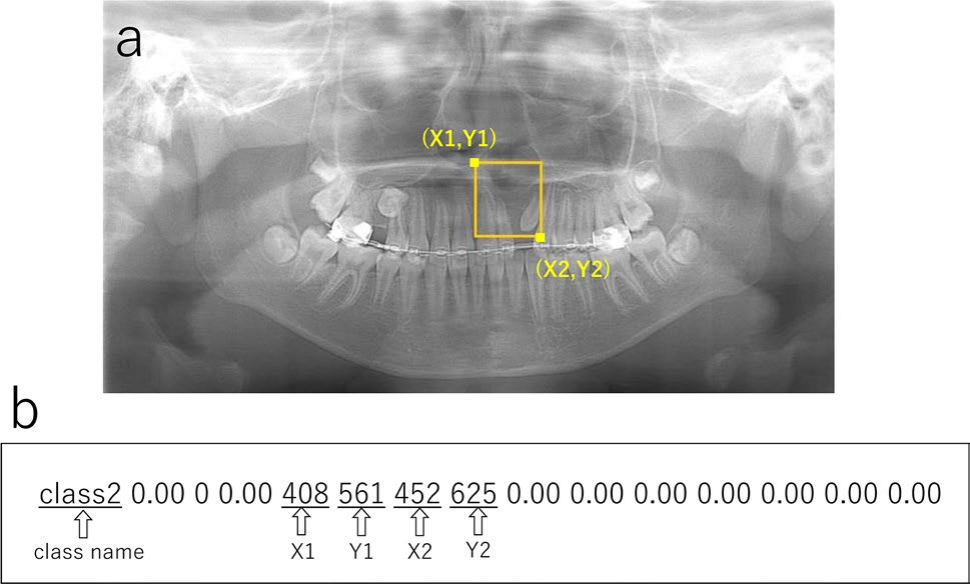
**(a)** The label including the cleft alveolus is created by setting a rectangular region of interest (ROI). The superior distal corner was set at the point including the equivalent level to the contralateral normal line of the piriform aperture’s lower limit and the most distal portion of the piriform aperture. The inferior medial corner was set at the alveolar ridge between the central incisors. **(b)** The coordinates of upper left and lower right corners of the ROIs were converted to text form.

In model 1, only two groups—CA only and CA with CP—comprised the training and validation sets (i.e., the normal group was not included). Rectangular regions of interest (ROIs) were set on the training and validation images to encompass the area of the CA according to the following methods. The superior margin was set at the level of the inferior line of the piriform aperture on the contralateral healthy side, and the inferior margin was set at the alveolar ridge. The medial end was set at the alveolar ridge between the central incisors, and the distal end was set at the most distal portion of the piriform aperture. The coordinates of the upper left (x1, y1) and lower right (x2, y2) corners of the ROIs were labeled using ImageJ (National Institute of Health, Bethesda, MD, USA) (Fig. 1a) and converted to text form (Fig. 1b). The CA only group and the CA with CP group were assigned as class 1 and class 2, respectively. Model 2 included the normal group’s data in addition to those of the patient groups. Only the labels were created for the classifications as class 0 (not the coordinates). In both models, 1000 epochs of the training process was performed. Inference was then applied to the test data, including all three groups, using the created learning models. When the model detected a CA, the detected area was shown as a bounding box. Red and blue boxes were shown for the CA only group and the CA with CP group, respectively.

### Comparison with diagnostic performance of human observers

To compare the DL model performances with those by human observers, two radiologists, who had more than four years experiences of interpretating panoramic radiographs, classified the same testing data, which were used to verify the DL performances, into one of three groups, namely, CA only, CA with CP, or Normal group.

### Analysis of image appearance features

To determine the characteristic image appearance features that could influence the DL models’ performance, the image datasets used for the training process (i.e., the training and validation datasets) were analyzed regarding two structures: the inferior line of the piriform aperture and the lateral incisor on the affected side. The former was evaluated based on its visibility and relative level to the contralateral or unaffected side. The latter was evaluated according to whether the tooth was present or absent, and regarding findings of microdontia, un-eruption and medial inclination. These evaluations were performed by two radiologists (YA and EA) with more than 20-years experiences of interpreting panoramic appearances. The final determinations were reached by consensus after discussion when the evaluations differed between the radiologists.

### Statistical analysis

The differences in ratios between two groups were tested by chi-square test, with p < 0.05 established as the threshold of significant difference.

## Results

**Figure 2:**
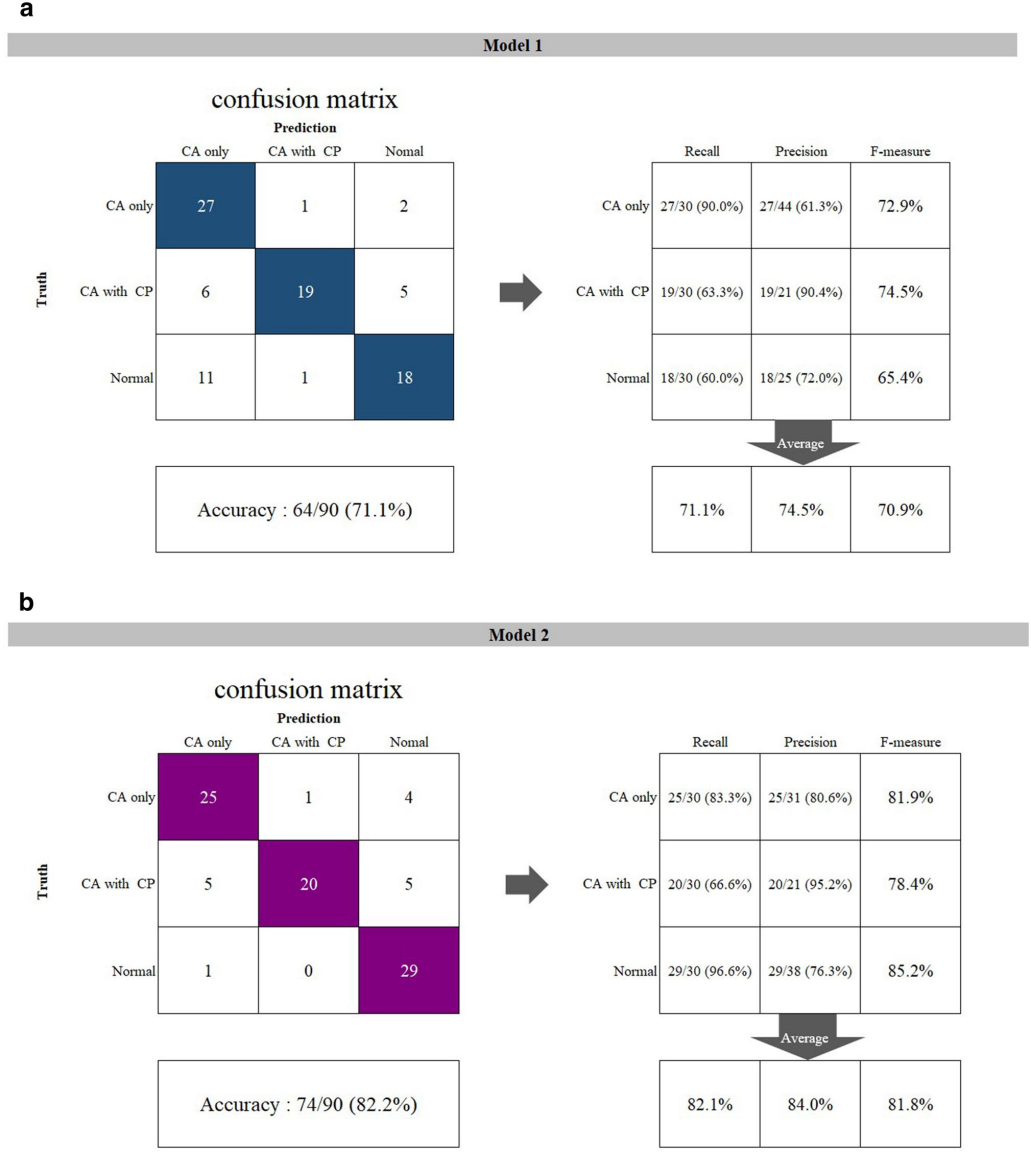

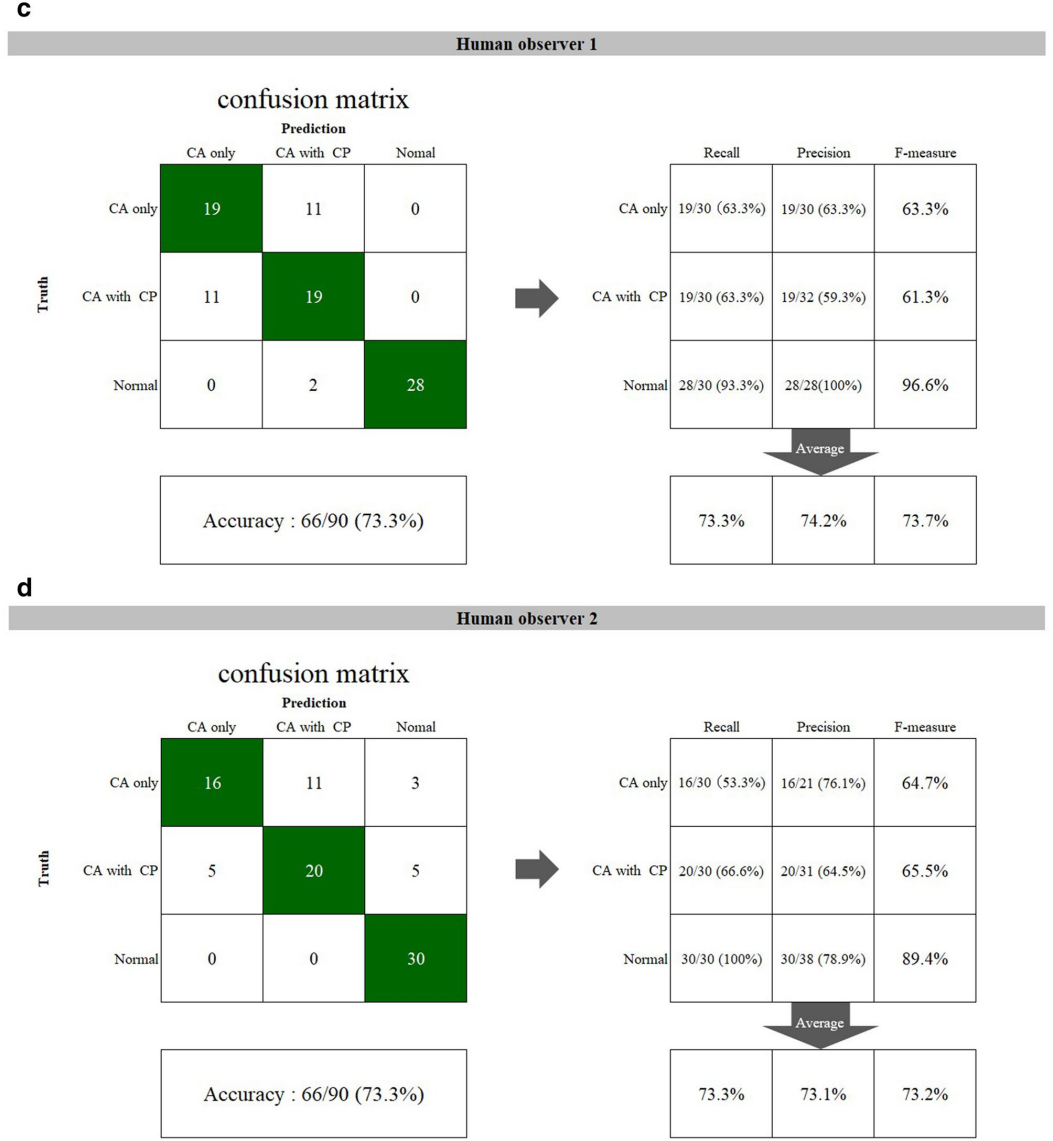
The confusion matrices of **(a)** model 1, **(b)** model 2, **(c)** human observer 1, and **(d)** human observer 2 and their calculated performance.

The results showed that no images had two or more bounding boxes on a panoramic image, indicating that only one box was detected per image when the model estimated a certain area as a cleft. All detected boxes sufficiently included the areas where a cleft was observed or would be in the normal group. Moreover, the boxes’ medial limit was not beyond the median line, and the distal limit was located medially to the canine. The superior and inferior limits were almost same as those of annotation area (namely, the inferior line of the piriform aperture and alveolar ridge). Therefore, we concluded that all test images could be predictively classified by both models into one of the three groups (CA only, CA with CP, or normal). Confusion matrix analyses were performed for both models (Fig. 2a,b), and the recall, precision, and F-measure values were calculated. The F-measure denotes the harmonic mean of recall and precision. Regardless of cleft status (CA only vs. CA with CP group), 53 (88.3%) and 51 (85.0%) of the 60 subjects who truly had a cleft could be detected by models 1 and 2, respectively. No difference was found between the models (p = 0.7883). Model 1 incorrectly assigned 12 (40%) of the 30 normal subjects as having a cleft, whereas only 1 normal subject was incorrectly assigned by model 2. No difference was found between models 1 and 2 in terms of the recall in the CA only group (p = 0.7041) or the CA with CP group (p = 0.7866), but the recall of the normal group was significantly higher in model 2 (p = 0.0017). Model 2 showed higher values of all three indices. The overall accuracy was higher in model 2 (82.2%) than model 1 (71.1%), but no significant difference (p = 0.0780) was found. The recall of the CA with CP group was poor (0.667) even in model 2.

Regarding human observers, confusion matrix analyses were also performed (Fig. 2c,d). Two observers showed a similar tendency for the performances. The overall accuracy of each human observer was higher than Model 1, however, lower than Model 2. The recall of Normal group was high, whereas those of the CA only group and the CA with CP group were poor in each human observer.

**Figure 3:**
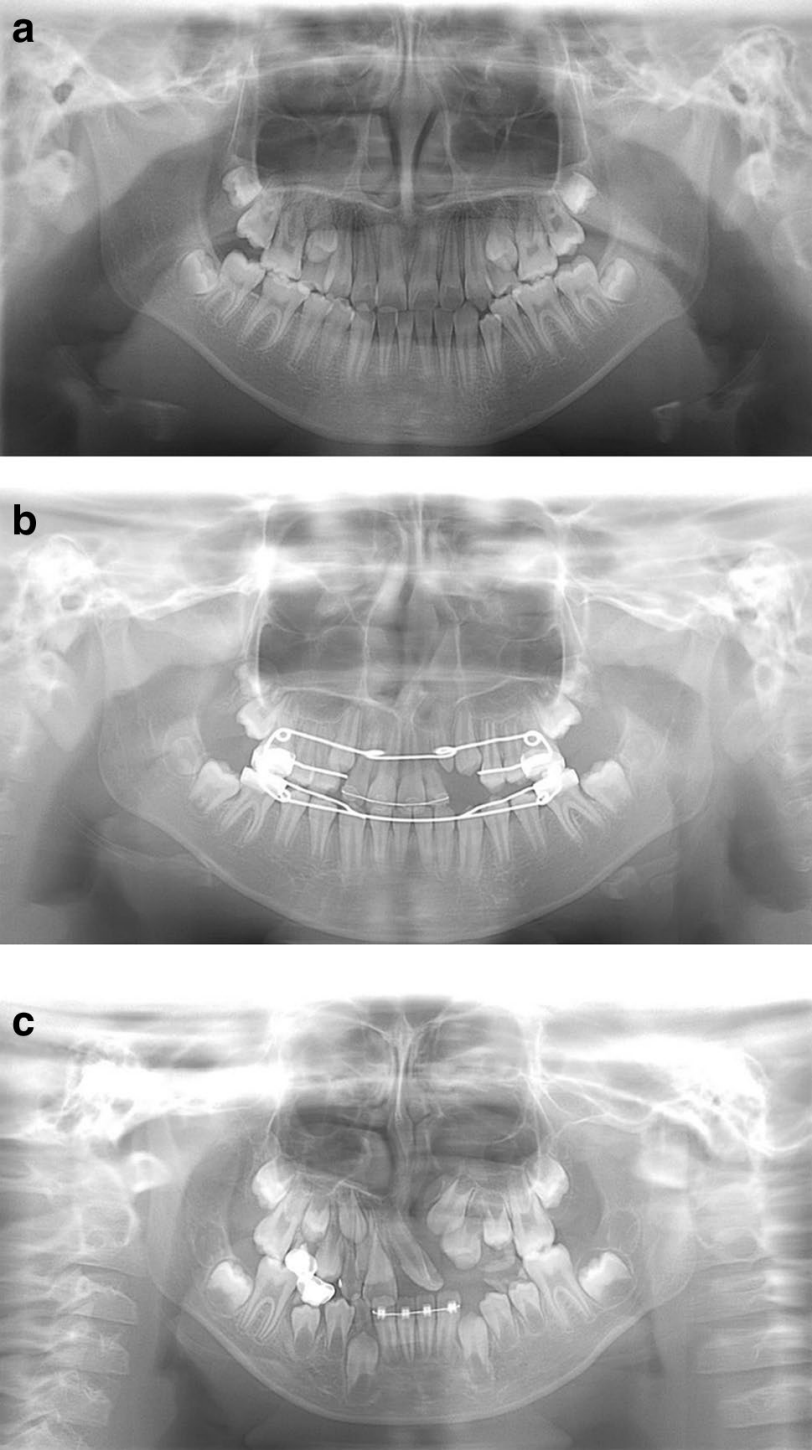
The findings of the inferior line of the piriform aperture were divided into three types. **(a)** is “clear and equivalent level”, **(b)** is “clear and inferior level”, and **(c)** is “obscure or invisible”.

The findings of the inferior line of the piriform aperture were divided into three types (Table 2, Fig. 3a–c).First, in 184 images, the lines could be observed clearly on the both right and left sides at an equivalent level and were assigned as “clear and equivalent level”. Second, 149 lines on the affected side were clearly visible but located at an inferior level relative to those on the unaffected side. This finding was defined as “clear and inferior level”. Third, 170 images were assigned as “obscure or invisible”, as they showed an obscure or invisible line on the affected side or on at least one side. The distributions of these three findings were significantly different among the three groups (p < 0.001). In the CA only group, most of the lines showed the finding of “clear and inferior level,” whereas the findings of “obscure or invisible” and “clear and equivalent level” were predominantly observed in the CA with CP and normal groups, respectively.

**Table 2:** Findings on the inferior line of the piriform aperture (number of images).

Group	Finding			
	Clear and equivalent level^a^	Clear and inferior level^b^	Obscure or invisible	Total
CA only	20 (13.9%)	108 (75.0%)	16 (11.1%)	144 (100%)
CA with CP	1 (0.6%)	41 (22.9%)	137 (76.5%)	179 (100%)
Normal	163 (90.6%)	0 (0.0%)	17 (9.4%)	180 (100%)

^a^The inferior line of the piriform aperture could be observed clearly on the both right and left sides at an equivalent level.

^b^The inferior line of the piriform aperture on the affected side was clearly visible but located at an inferior level relative to the unaffected side, or the line on a side was located at an inferior level relative to the contralateral side in normal subjects.

The findings of the lateral incisor on the affected side are summarized in Table 3. The distributions of all four findings were significantly different among the three groups (p < 0.001). The observation rates of all findings were low in the normal group relative to those in the CA only and CA with CP groups.

**Table 3:** Findings of the lateral incisor on the affected side (number of images).

Group	Appearance			
	Absence*	Microdontia*	Unerupted*	Medial inclination*
CA only	51/144 (35.4%)	75/144 (52.1%)	68/144 (47.2%)	16/144 (11.1%)
CA with CP	77/179 (43.0%)	81/179 (45.3%)	77/179 (43.0%)	33/179 (18.4%)
Normal	19/180 (10.6%)	0/180 (0.0%)	5/180 (2.8%)	0/180 (0.0%)

*Difference between distributions is significant among three groups (p < 0.001).

**Figure 4:**
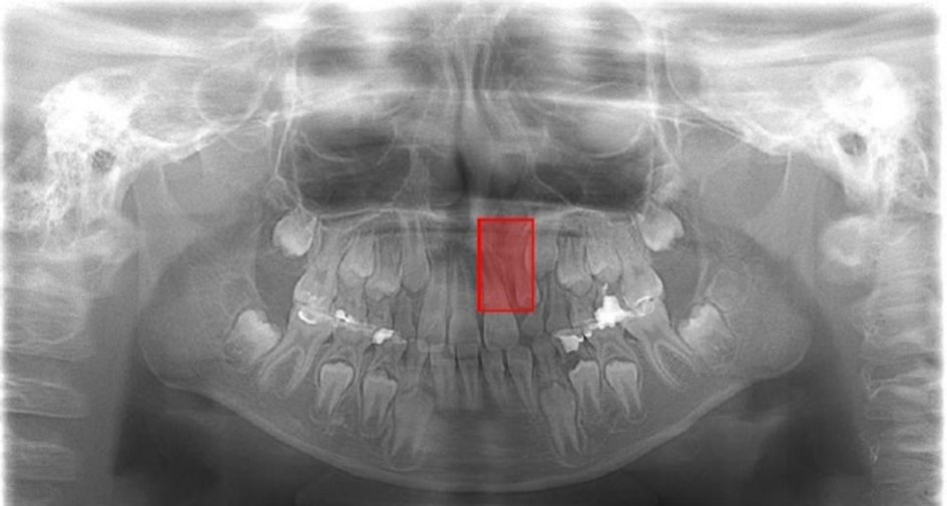
A correctly detected and classified case. In a patient belonging to the CA only group, the left side CA was correctly detected and classified into the CA only group, showing a red bounding box. The inferior line of the piriform aperture on the affected side is situated at an inferior level relative to that on the contralateral normal side. The left lateral incisor shows findings of unerupted microdontia.

**Figure 5:**
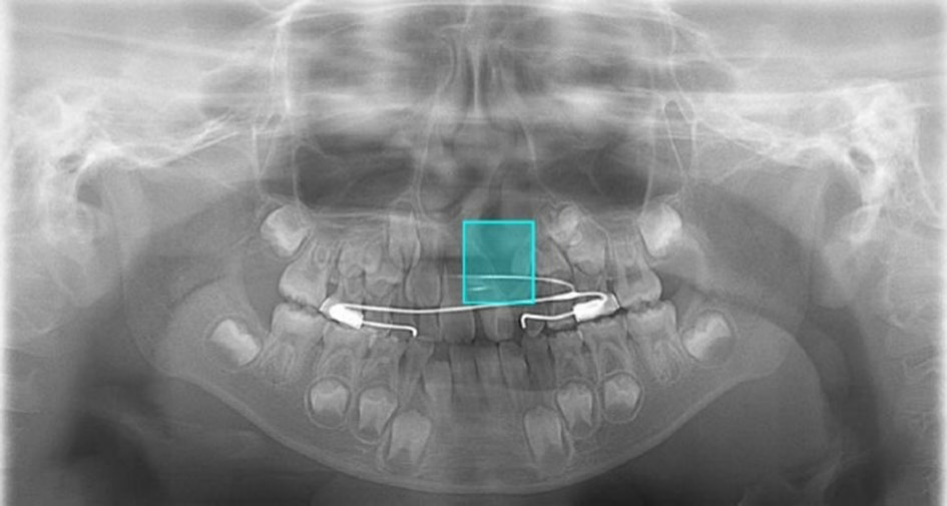
A correctly detected but erroneously classified case. On a panoramic image of a patient with solely CA (CA only group), the cleft area was correctly detected but is misclassified into the CA with CP group. The lateral incisor shows findings of microdontia with slight medial inclination, but the inferior piriform aperture line is obscure on the affected side, and the radiolucency of the cleft area is relatively high.

**Figure 6:**
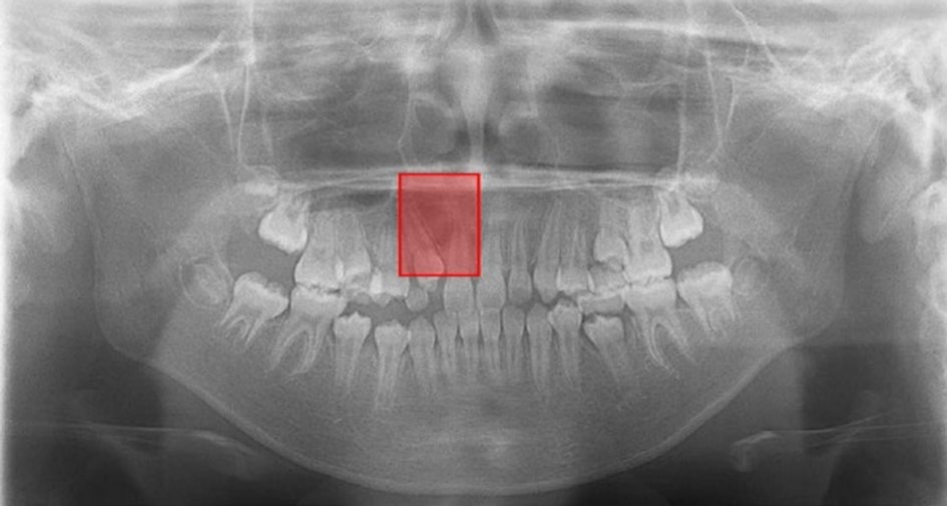
A falsely detected normal case. In a normal subject (without CA), a red bounding box is shown on the right incisor region. The inferior piriform aperture lines on the both sides are obscure and superimposed by the radiopaque lines caused by the hard palate. The right lateral incisor is missing (probably congenitally), and the canine shows medial inclination, so the space between these teeth is relatively wide. The model probably mistakenly recognizes the canine as the lateral incisor.

**Figure 7:**
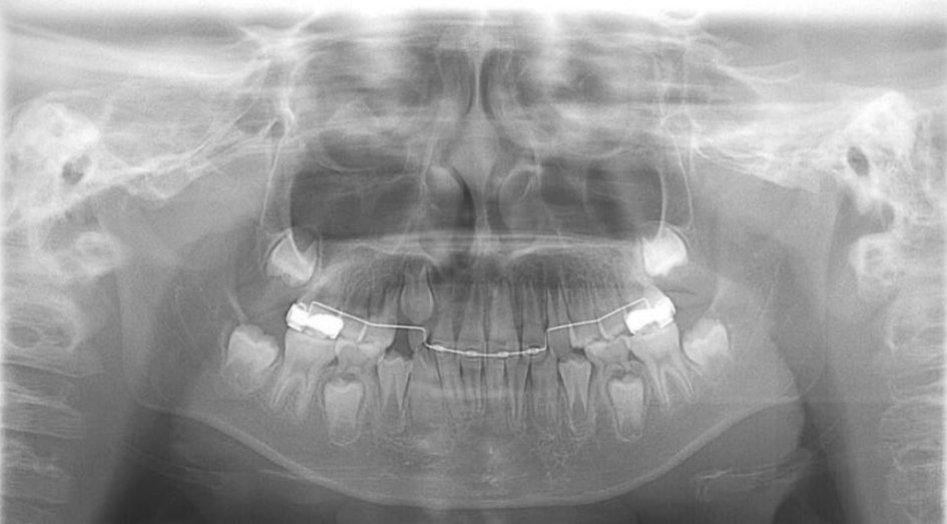
An undetected case with CA. The panoramic image of a subject with CA (CA only group) on the right side shows no bounding box. The inferior piriform aperture lines are observed at almost equal levels, and the right lateral incisor shows no characteristic findings.

Examples of model-predicted results are shown in Figs. 4, 5, 6 and 7. Figure 4 shows a result from the CA only group with a correctly detected and correctly classified bounding box. The result in Fig. 5 was correctly detected as having CA but falsely classified into the CA with CP group. Figure 6 shows a falsely detected area in a normal group subject. In Fig. 7, the cleft could not be detected.

## Discussion

The detection function of DL systems on panoramic radiographs has been studied for various lesions and conditions^20–27^. Regarding vertical root fracture^20^ and dental implants^21^, detection models have been created with annotations of only fractured teeth or implant sites. Normal teeth without fractures or replacement by implants were not included in the training, validation, or test datasets, probably because many normal teeth were included in each panoramic image. Several authors reported relatively high detection sensitivity (recall in the present study) for maxillary and mandibular cyst-like lesions^22–25^. Ariji et al.^22^ and Watanabe et al.^23^ created and tested their DetectNet models without annotation of normal subjects’ data. Hyunwoo et al.^26^ and Odeuk et al.^27^ built models using the You Only Look Once system without normal subjects’ data and tested them with datasets including normal subjects without cyst-like lesions. In all of those studies, the training and validation processes were performed without normal subjects. Therefore, we created a model without normal subjects in addition to the model developed with normal subjects’ data, whereas both models’ test datasets included normal subjects. Model 1’s results showed that 12 (40%) of the 30 normal subjects in the test set were falsely assigned as having a cleft, whereas only one of those subjects was assigned to the CA only group by model 2 (which included the normal subjects in the learning process). Although the cause of this discrepancy between cyst-like lesions and CAs could not be completely elucidated, the characteristics of the lesions’ appearances might contribute to the difference. Cyst-like lesions showed definite borders on radiographs, but cleft areas were frequently represented as ill-defined radiolucency because of the superimposition of surrounding structures, such as unerupted permanent teeth.

The present results showed relatively high detection sensitivity (recall in the present study) compared with the corresponding values of other studies on panoramic radiographs^22,23^, regardless of the cleft status for which the values were determined (CA only or CA with CP groups). However, even model 2 had a low recall value of 0.667 for the CA with CP group. This problem should be solved in future investigation and could be accomplished by increasing the size of the training dataset.

Regarding human observers, the recall of normal group was high, however, overall accuracy was lower than Model 2. This result suggested the deep learning model, which included the normal group for learning process, had the potential to assist the human observers as a CAD system.

Although the most important feature for CA detection might be radiolucent area at the maxillary incisor region, the frequently reported characteristic appearance other than radiolucent area, such as findings of the lateral incisor on the affected side and of the inferior line of the piriform aperture, were analyzed to clarify potential features related to the created models’ performance. Regarding the inferior line of the piriform aperture, Hansen et al.^12^ reported that the line was positioned 2.9 mm lower on the cleft side than on the noncleft side, whereas no difference was noted between the right and left sides in the normal group (mean difference: <1 mm). Therefore, based on the visibility and the line level, we analyzed the appearance with >2 mm difference defined as abnormal. Consequently, the appearance was divided into three types (clear and inferior level, obscure or invisible groups, and clear and equivalent level), and the ratios were 75%, 76%, and 90% for the CA only, CA with CP, and normal groups, respectively. This result suggests that the findings of the inferior line of the piriform aperture might be related to both classification performance and detection performance. Regarding the lateral incisor, microdontia were observed in 48% of patients with CA, but not in any patients in the normal group. Therefore, the presence of microdontia might be related to the model’s detection performance of CA. Similarly, unerupted lateral incisors were observed in 44% of patients with CA but only 2.7% of patients in the normal group, suggesting that this factor is also related to the models’ performance.

The present study had several limitations. First, patients with bilateral CA were excluded because it was difficult to clearly visualize the inferior line of the piriform aperture in such patients’ panoramic images. This would result in failure of the determination of the superior limit of the ROIs for the learning process. Future study should be conducted to address these patients. In this regard, it might be effective to test the model created in the present study by using the panoramic radiographs of patients with bilateral CAs. Second, the numbers of training and test data were so small that the results cannot be generalized although it was difficult to estimate an appropriate sample size ^28^. Future research should be planned with larger datasets obtained from multiple hospitals through the use of different panoramic machines. Third, other CNNs and functions, such as semantic segmentation, should be used to improve the model’s performance.

## Conclusions

The model developed in the present study appears to have the potential to detect CAs and classify them into CA only and CA with CP groups on panoramic radiographs, which may be useful as assisting the performance of human observers. Additionally, some performance-related differences between three experimental groups (CA only, Ca with CP and normal groups) were clarified.
